# The External Validation of GLOBE and UK-PBC Risk Scores for Predicting Ursodeoxycholic Acid Treatment Response in a Large U.S. Cohort of Primary Biliary Cholangitis Patients

**DOI:** 10.3390/jcm13154497

**Published:** 2024-08-01

**Authors:** Ana Marenco-Flores, Natalia Rojas Amaris, Tamara Kahan, Leandro Sierra, Romelia Barba Bernal, Esli Medina-Morales, Daniela Goyes, Vilas Patwardhan, Alan Bonder

**Affiliations:** 1 Division of Gastroenterology, Hepatology, and Nutrition, Beth Israel Deaconess Medical Center, Harvard Medical School, Boston, MA 02215, USA; amarenco@bidmc.harvard.edu (A.M.-F.); nrojasam@bidmc.harvard.edu (N.R.A.); vpatward@bidmc.harvard.edu (V.P.); 2 Department of Internal Medicine, Texas Tech University System, Lubbock, TX 79430, USA; 3 Department of Medicine, Rutgers New Jersey Medical School, Newark, NJ 07103, USA; 4 Division of Digestive Diseases, Yale School of Medicine, New Haven, CT 06520, USA

**Keywords:** biliary, cholangitis, prognosis, ursodeoxycholic acid, prognosis, GLOBE, UK-PBC

## Abstract

**Background:** The cornerstone treatment for primary biliary cholangitis (PBC) is ursodeoxycholic acid (UDCA), but many patients exhibit an incomplete response, leading to disease progression. Risk prediction models like the GLOBE and UK-PBC scores hold promise for patient stratification and management. We aimed to independently assess the predictive accuracy of these risk scores for UDCA response in a prospective U.S. cohort. **Methods:** We conducted a prospective cohort study at a U.S. liver center, monitoring UDCA-treated PBC patients over a one-year follow-up. We evaluated the predictive efficacy of the GLOBE and UK-PBC scores for UDCA treatment response, comparing them to the Paris II criteria. Efficacy was assessed using univariate and multivariate analyses, followed by prognostic performance evaluation via receiver operating characteristic (ROC) curve analysis. **Results:** We evaluated 136 PBC patients undergoing UDCA therapy. Based on the Paris II criteria, patients were categorized into UDCA full-response and non-response groups. The GLOBE score identified a non-responder rate of 18% (*p* = 0.205), compared to 20% (*p* = 0.014) with the Paris II criteria. Multivariate analysis, adjusted for age and biochemical markers, showed that both the GLOBE and UK-PBC scores were strongly associated with treatment response (*p* < 0.001). The area under the ROC curve was 0.87 (95% CI 0.83−0.95) for the GLOBE score and 0.94 (95% CI 0.86−0.99) for the UK-PBC risk score. **Conclusions:** Our study demonstrates that GLOBE and UK-PBC scores effectively predict UDCA treatment response in PBC patients. The early identification of patients at risk of an incomplete response could improve treatment strategies and identify patients who may need second-line therapies.

## 1. Introduction

Primary biliary cholangitis (PBC) is a chronic, progressive cholestatic liver disease characterized by the autoimmune destruction of intrahepatic bile ducts. The estimated global incidence and prevalence rates of PBC are 1.76 and 14.60 per 100,000 persons, respectively [1]. In the United States (U.S.), the annual incidence of PBC is higher, at 2.75 per 100,000 persons, compared to Europe at 1.86, with the lowest incidence observed in the Asia–Pacific region at 0.84 [2]. PBC predominantly affects females (>90%) and is typically diagnosed in the fourth or fifth decade of life [3]. PBC is a progressive disease; without treatment, it advances to end-stage liver disease. A prospective study by Christensen et al. revealed that untreated PBC patients exhibited histologic progression within two years [4]. The risk of developing decompensated cirrhosis has been estimated at 15% to 25% over five years [5].

Treatment with ursodeoxycholic acid (UDCA) improves liver biochemistry, slows hepatic fibrosis progression, and may extend life expectancy [6]. UDCA has transformed the treatment landscape for PBC, with transplant-free survival rates of 79.7% among treated patients compared to 60.7% among untreated patients [7]. However, a substantial proportion of patients exhibit an inadequate response to UDCA, leading to a higher risk of liver-related disease progression [8]. Several criteria for evaluating UDCA treatment response have been developed, including the Rotterdam, Barcelona, Rochester-II, Paris, Toronto, and Ehime criteria. These prognostic models assess therapeutic effects based on liver biochemical parameters after 6, 12, or 24 months of UDCA treatment [9]. Despite their utility, these criteria have limitations, particularly for patients with inadequate responses, as they may continue ineffective treatment for extended periods, increasing the risk of disease progression [10].

Two independent research groups, the Global PBC Study Group and the United Kingdom (UK)-PBC Consortium, have developed and externally validated continuous prognostic models: the GLOBE and UK-PBC risk scores, respectively [11,12]. The GLOBE score was designed to estimate the risk of liver transplantation (LT) or overall death in patients with PBC who have been treated with UDCA for one year [10]. A previous retrospective study by Harms et al. estimated the score for both patients treated with UDCA and those who were not, evaluating LT-free survival [7]. Additionally, changes in the GLOBE score from baseline to one year after the initiation of UDCA and from one year to two years after the initiation of UDCA have been associated with LT or death [13]. Similarly, the UK-PBC risk score was developed to predict the risk of developing end-stage liver disease in patients treated with UDCA [12] and has been validated in North American cohorts [14]. Although both scores were originally validated in a Western population, a study from China demonstrated that these scores also provide reliable estimates in diverse ethnic populations [15].

Previously, single-center external validations of the GLOBE and UK-PBC scores within the U.S. included a validation study of the UK-PBC score at the Mayo Clinic, which involved a 20-year retrospective cohort of 464 patients with PBC [14]. Additionally, a retrospective study at the Cleveland Clinic evaluated both the GLOBE and UK-PBC scores in 352 PBC and PBC/overlap patients treated with UDCA between 1998 and 2017 [16]. Our study represents the largest prospective cohort study conducted at a tertiary center in the U.S., focusing exclusively on patients with PBC-only diagnoses.

Risk prediction models such as the GLOBE and UK-PBC scores play a crucial role in informing decision-making and guiding future patient management. It is essential that these models demonstrate transferability and can be confidently applied across diverse patient populations with PBC. Therefore, robust validation in external patient cohorts is essential before integrating these models into clinical practice. In this study, we aimed to independently evaluate the predictive performance of the GLOBE and UK-PBC risk scores in response to UDCA treatment in a prospective cohort in the United States.

## 2. Materials and Methods

### 2.1. Study Population

The subjects for this study were enrolled in a prospective autoimmune liver registry at Beth Israel Deaconess Medical Center (BIDMC; Boston, MA, USA). Enrollment occurred between January 2018 and November 2023. Subjects were eligible if they met the PBC diagnosis criteria based on internationally accepted standards, as recommended by the European Association for the Study of the Liver (EASL). Diagnosis of PBC was confirmed by the presence of at least two of the following criteria: (a) elevated alkaline phosphatase (ALP) levels; (b.1) the presence of antimitochondrial antibody (AMA) at a titer >1:40; or (b.2) the presence of anti-sp100/anti-glycoprotein 210 (anti-gp210); or (c) in cases of AMA-negative subjects, a liver biopsy showing classic histologic findings of PBC.

**Figure 1:**
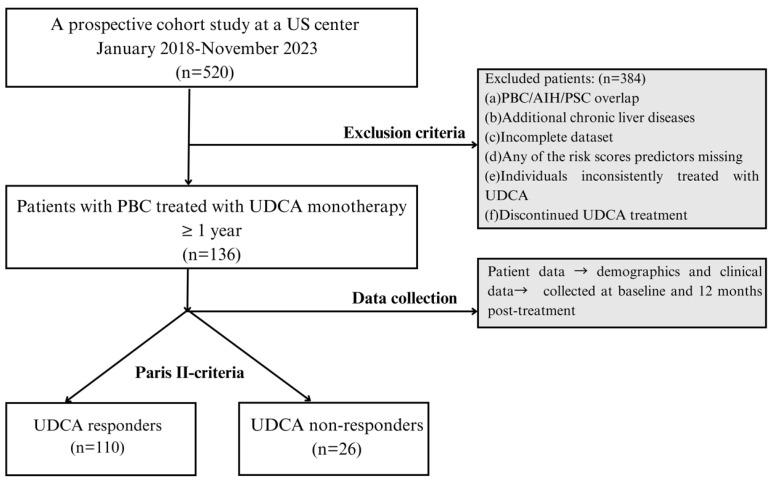
Flowchart of cohort selection. AIH, autoimmune hepatitis; N, number; PBC, primary biliary cholangitis; PSC, primary sclerosing cholangitis; UDCA, ursodeoxycholic acid.

The exclusion criteria were as follows: age under 18 years at study entry, the presence of any autoimmune overlap syndrome, history of concomitant liver disease, missing data that prevented the assessment of treatment response, missing predictors for any of the risk scores, the absence of UDCA treatment or an unknown date of initial UDCA treatment, and the discontinuation of UDCA treatment within the first year (Figure 1). Ultimately, we analyzed 136 adult PBC patients who met the specified cohort characteristics.

### 2.2. Study Outcome, Variables, and Definitions

The study’s outcome was the response to UDCA therapy, defined by the Paris II criteria as ALP levels ≤ 1.5 times the upper normal limit (ULN), AST levels ≤ 1.5 times the ULN, or bilirubin levels < 1 mg/dL after one year of treatment. In this study, patients received the recommended UDCA dose (13 to 15 mg/kg).

Patient data extracted from electronic medical records included age at diagnosis, gender, body mass index (BMI), initial UDCA dose, and diagnostic studies (AMA positivity, elevated ALP, and histologic findings). Additionally, baseline laboratory results, including ALP, alanine transaminase (ALT), aspartate aminotransferase (AST), total bilirubin (TB), albumin (ALB), platelets (PL), white blood cell count (WBC), prothrombin time (PT), international normalized ratio (INR), and immunoglobulin G (IgG), were collected at treatment initiation. Following 12 months of UDCA therapy, data on ALP, ALT, AST, TB, ALB, PL, PT, and INR were collected for analysis.

We evaluated the predictive efficacy of the GLOBE score for UDCA treatment response, comparing it to the Paris II criteria. Patients with a GLOBE score above 0.30 were classified as non-responders, whereas those with a GLOBE score of 0.30 or less were classified as responders [11]. The GLOBE score was calculated using the following equation:

GLOBE score = 0.044378 × age at start of UDCA therapy + 0.93982 × ln (TB times the upper limit of normal [ULN] at 1 year follow-up) + 0.335648 × ln (ALP × ULN at 1 year follow-up) − 2.266708 × ALB level × the lower limit of normal (LLN) at 1 year follow-up −0.002581 × PL count per 109/L at 1 year follow-up +1.216865(1)

Subsequently, we assessed the 5-, 10-, and 15-year risks using the UK-PBC risk score at baseline and 12 months post-UDCA treatment to observe the score’s evolution over time. The UK-PBC risk scores were calculated using the equation provided by the UK-PBC Project, as follows:

UK-PBC risk score = 1 − baseline survival function∧ exp(0.0287854 × [ALP baseline and after 12 months of therapy × ULN − 1.722136304] − 0.0422873 × [{(ALT where this was available, otherwise AST, baseline and after 12 months of therapy × ULN/10)∧−1} − 8.675729006] + 1.4199 × [ln{TB after 12 months of therapy × ULN/10} + 2.709607778] − 1.960303 × [ALB at baseline × LLN − 1.17673001] − 0.4161954 × [PL count at baseline × LLN − 1.873564875]).(2)

### 2.3. Statistical Analysis

Demographic, clinical, and biochemical markers were collected, presenting continuous variables with a normal distribution as mean and standard deviation (SD) and non-normal variables as median and interquartile range (IQR). Comparisons were made using a *t*-test or Mann–Whitney U test, as appropriate. Categorical variables, summarized as percentages, were compared using Pearson’s chi-squared test (χ^2^). 

For the assessment of predictors of treatment effectiveness, we employed logistic regression models. The preliminary univariate model included potential covariates at index (age and laboratory results for ALP, ALT, AST, TB, ALB, PL, GLOBE, and UK-PBC scores). Variables with *p* values < 0.05 were retained in the multivariate model, and results are presented as odds ratios (ORs) with 95% confidence intervals (CIs), with statistical significance defined as *p* < 0.05. 

The predictive performance was assessed by calculating and plotting the area under the receiver operating characteristic curve (AUROC) and estimating the 95% CI for each risk score.

All statistical analyses were conducted using Stata version 18.0 (StataCorp LP, College Station, TX, USA).

## 3. Results

### 3.1. Baseline Characteristics

Our study included 136 patients diagnosed with PBC who had been on continuous UDCA therapy for a year. The baseline characteristics of the cohort are detailed in Table 1. The majority of patients were female (90%) and predominantly white Caucasian (79.0%), with a mean age of 56 years. For PBC diagnosis, 82% of patients met ALP criteria, 63% had positive antibodies, and 51% had compatible liver biopsies. Additionally, 12.5% of patients already had cirrhosis at their initial visit.

### 3.2. Treatment Response

At the 12-month follow-up post-UDCA treatment, as shown in Table 2, liver function-associated biomarkers—ALP, ALT, AST, TB, and PL—showed significant differences in the UDCA non-responding group compared to the complete-response group (*p* < 0.05 in all analyses). In contrast, the albumin levels in the UDCA non-responding group remained comparable to those in the complete-response group (*p* = 0.001). At the end of follow-up, responder PBC patients exhibited a mean GLOBE of −0.82 (SD = 1.02). GLOBE score performance, compared to Paris II criteria, indicated a non-responder rate of 18% (*p* = 0.205) and 20% (*p* = 0.014), respectively.

Baseline UK-PBC scores revealed lower mean scores in responders compared to non-responders across 5-, 10-, and 15-year risk calculations (mean 1.12 vs. 3.29; 3.17 vs. 10.12; 5.26 vs. 17.27, *p* < 0.001). Following 12 months of UDCA treatment, significant changes in scores accentuated this difference, with responders consistently displaying lower mean scores compared to non-responders across 5-, 10-, and 15-year risk calculations (mean 0.52 vs. 2.44; 1.70 vs. 7.78; 3.13 vs. 13.65, *p* < 0.001) (Table 3). 

Our univariate analysis revealed significant associations between treatment response and levels of ALP (OR, 1.02; 95% CI, 1.00–1.05; *p* = 0.027) and AST (OR, 1.02; 95% CI, 1.00–1.04; *p* = 0.006), TB (OR, 9.78; 95% CI, 5.82–13.7; *p* < 0.001), as well as both risk scores: GLOBE (OR, 6.64; 95% CI, 3.13–14.05; *p* < 0.001) and UK-PBC scores (OR, 19.3; 95% CI, 5.98–62.5; *p* < 0.001) (Table 4). 

The logistic regression multivariate analysis, adjusted for age and biochemical markers, demonstrated a strong association between the GLOBE score and PBC responders to UDCA treatment (OR 9.30, 95% CI 3.21–24.9, *p* < 0.001). Similarly, there was a significant association between the UK-PBC risk score and PBC responders to UDCA treatment (OR 32.97, 95% CI 7.44–45.97, *p* < 0.001).

### 3.3. Predictive Performance

**Figure 2:**
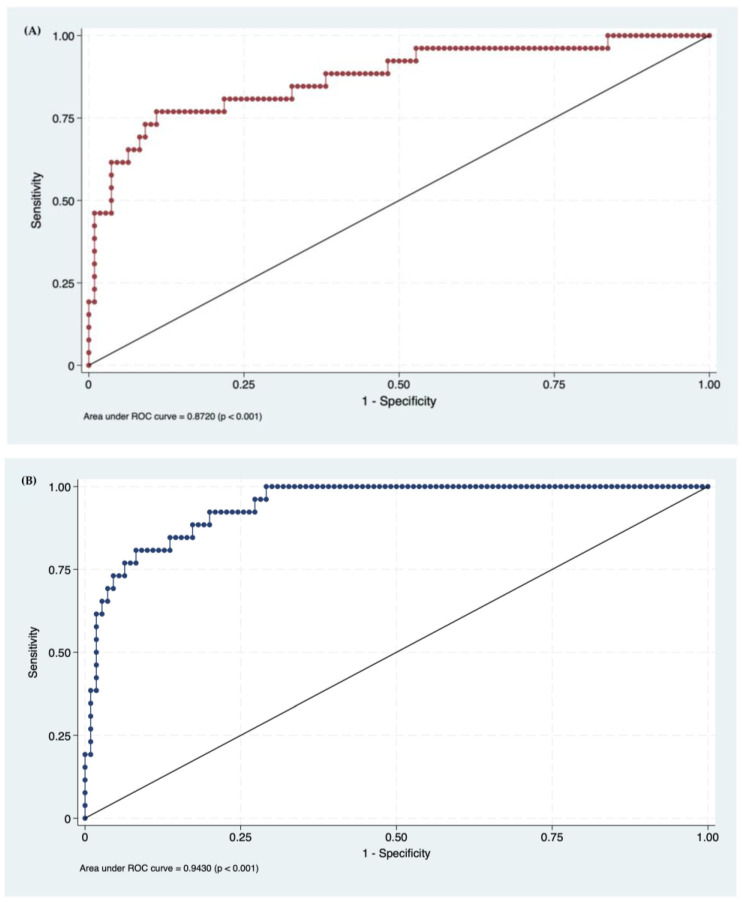
Predictive performance of risk scores. (**A**) AUROC GLOBE score 0.87 (CI 95% 0.83–0.95, *p* < 0.001) (**B**) AUROC UK-PBC score 0.94 (CI 95% 0.86–0.99, *p* < 0.001).

We confirmed the high discrimination ability of both the GLOBE (AUROC 0.87; 95% CI 0.83−0.95, *p* < 0.001) and UK-PBC risk score models (AUROC 0.94; 95% CI 0.86−0.99, *p* < 0.001) for treatment response in a single-center U.S. patient cohort (Figure 2).

## 4. Discussion

Our findings validate the utility of both the GLOBE and UK-PBC risk scores in accurately predicting treatment response in PBC. The GLOBE score, proposed by Lammers et al. [11], was developed using a derivation cohort of 2488 cases and a validation cohort of 1634 patients. This score combines predictive information on disease severity and treatment response. Initially designed to estimate the risk of death or LT after one year of UDCA therapy, recent studies indicate that the GLOBE score can also stratify UDCA-treated patients beyond that period [17].

Approximately 40% of PBC patients exhibit an incomplete response to UDCA, resulting in a worse prognosis compared to responders [3]. Moreover, other studies have reported that 20–30% of PBC patients exhibit incomplete biochemical responses to UDCA, highlighting the benefits of individualized treatment plans and personalized management strategies [18,19,20]. An international cohort study validated the GLOBE score using data from the Global PBC Study Group, which included patients from eight countries in Europe and North America [7]. Among 3433 patients treated with UDCA, 733 (21.4%) were classified as inadequate responders one year after initiating UDCA therapy. Similarly, our results showed that 23 patients (18%) were classified as non-responders using the GLOBE score. In comparison, the Paris criteria identified 26 patients (20%) with an inadequate response to treatment. These results were expected, as the Paris II criteria consider three biochemical parameters, while the GLOBE score includes a broader range of parameters [21].

The lower percentage of non-responders to UDCA treatment in these study cohorts can be attributed to several factors supported by the existing literature. Firstly, our study cohort consisted of a significant proportion of subjects with early PBC, as it was developed for a prospective registry with a large proportion of patients enrolled at diagnosis. Hirschfield et al. [22] suggest that the early initiation of UDCA treatment, particularly within the first two years of diagnosis, is associated with improved response rates and slower disease progression. Furthermore, we only included patients who met the inclusion criteria, which were more stringent than those of other validation cohorts.

Despite their importance as prognostic markers, hepatic transaminases are not included in the GLOBE score. Our study found that AST and ALT were not significantly associated with an incomplete response to UDCA treatment, as confirmed by multivariate analysis. Previous research supports these findings. Mane et al.’s retrospective study of 53 PBC patients treated with UDCA for one year showed a significant reduction in ALP but no significant decrease in paired AST and bilirubin levels [23]. Similarly, Cortez-Pinto et al. found that ALT levels and increased bilirubin were not associated with an incomplete response [21]. Additionally, our findings demonstrated a significant association between ALP levels and treatment response, which is supported by Lammers et al.’s meta-analysis. This analysis demonstrated a log-linear relationship between ALP levels and LT-free survival, indicating that lower alkaline phosphatase values correlate with longer LT-free survival [24].

Our study found that age was not significantly associated with an incomplete response to treatment, consistent with findings from a previous Portuguese observational cohort study of 434 PBC patients assessing UDCA treatment response [21]. Previous studies have indicated that younger patients at diagnosis have a higher risk of treatment failure, likely due to presenting with a more severe form of the disease, potentially associated with a ductopenic phenotype resistant to UDCA therapy [25]. In contrast, another study identified older age at diagnosis as an independent predictor of mortality in PBC patients [26].

The one-year follow-up design and the inclusion criteria of this study limit the assessment of long-term hepatic decompensation events, which were not evident in the definitive cohort. A retrospective study by Gazda et al., including 249 Slovakian patients, primarily aimed to evaluate the risk of hepatic decompensation after UDCA therapy by assessing prognostic factors in PBC over a ten-year span. The study demonstrated that treatment failure after six months of UDCA therapy is linked to a 12-fold increase in the risk of liver decompensation, including ascites, hepatic encephalopathy, or variceal bleeding. Additionally, treatment failure after twelve months of UDCA therapy is associated with a 22-fold increase in the risk of liver decompensation [27].

The GLOBE score is an essential tool for managing PBC patients, providing an effective assessment of both treatment response and the risk of adverse outcomes [11,28]. Data from the Global PBC cohort indicate that changes in the GLOBE score during the first and second years predict death/LT-free survival, with hazard ratios (HRs) of 2.28 (*p* < 0.001) and 2.19 (*p* < 0.001), respectively, independent of the baseline score [29]. These findings suggest that monitoring the GLOBE score at multiple time points can enhance the accuracy of outcome prediction [28]. Additionally, a recent study by Montano et al., including 332 patients with recurrent PBC after LT from 28 centers across Europe, North America, and South America, demonstrated that both the GLOBE score and the UK-PBC score can identify patients at higher risk of graft loss and mortality post-LT [30].

The UK-PBC score, developed by Carbone et al., is based on a large cohort of 1916 British patients and has been further validated in an independent cohort of 1249 patients [12]. This risk score allows for the accurate long-term prediction of LT and liver-related death over 5, 10, and 15 years, with an AUROC exceeding 0.90. Our study examined the UK-PBC score as a prognostic tool for treatment response and revealed temporal variability in overall risk before and after UDCA treatment. This improvement in prognostic scores following UDCA treatment aligns with previous studies correlating biochemical response with survival outcomes. A study of 192 PBC patients treated with UDCA demonstrated that a good biochemical response after one year is associated with survival rates similar to those of a matched control population, highlighting the beneficial effects of UDCA in PBC [31]. Additionally, a systematic review and meta-analysis by Gazda et al. showed that the HR of the continuous UK-PBC risk score was 3.39 for liver events (95% CI: 3.10–3.72), while the HR of the binary form was 2.76 (95% CI: 2.14–3.69) [32]. These findings support the utility of the UK-PBC score in predicting long-term outcomes and the effectiveness of UDCA treatment in PBC.

The original studies highlighted the excellent predictive abilities of the GLOBE and UK-PBC risk scores [11,12]. Our study cohort also demonstrated comparable and highly accurate results across overall risk scoring systems, with the GLOBE score having an AUROC greater than 0.87 and the UK-PBC score having an AUROC greater than 0.94. The high discriminative performance of the UK-PBC risk score suggests the effective categorization of individuals based on their likelihood of responding to treatment [33]. Several factors contribute to the excellent performance of these models. First, the GLOBE and UK-PBC scores incorporate multiple key independent variables such as age, bilirubin, ALP, albumin, and platelet count, unlike other criteria that solely focus on treatment response and may not account for cirrhosis. Additionally, most other models rely on laboratory data collected one year after starting UDCA treatment, while the GLOBE and UK-PBC scores use laboratory values from two time points (baseline and one year after starting UDCA), enhancing their precision [33,34]. Thus, the dichotomization of continuous variables in previous models can impact their robustness [33].

The prospective design of this study has inherent limitations. The primary constraint is the size of our cohort. Conducting large single-center prospective studies on PBC is challenging due to the disease’s low prevalence and slow progression. Additionally, we excluded patients with incomplete information, which may have introduced selection bias. Furthermore, other clinical events that arise during PBC may interfere with the performance of risk scores. Finally, while our results confirm the predictive ability of the UK-PBC and GLOBE risk scores for treatment response, they do not address the calibration of these scores, as our outcome definition differs from that of the original studies.

While we acknowledge the study’s limitations, these challenges underscore the importance of extended observation periods for a more comprehensive understanding of PBC patients. Future research should prioritize validating prognostic factors through a combination of retrospective and prospective studies, evaluating their contribution to newly developed prediction models.

## 5. Conclusions

Our study demonstrated the good prognostic performance of the GLOBE and UK-PBC scores in predicting UDCA treatment response for PBC patients. The early identification of patients at risk of an incomplete response could enhance treatment strategies. Consequently, these response criteria are crucial for selecting patients who may require additional second-line therapies.

## Figures and Tables

**Table 1:** Baseline characteristics and demographics (n = 136).

Variables	Baseline (N = 136)
Age, mean (SD)	56 (12)
Gender, female N (%)	123 (90)
Race, N (%)	
White Caucasian	107 (79)
Hispanic or Latino	11 (8)
African American	1 (0.74)
Other/multiracial	8 (6)
Asian	9 (7)
BMI, mean (SD)	29 (6)
Diagnosis method	
Elevated ALP, N (%)	111 (82)
Positive AMA/positive sp100, anti-gp210, N (%)	86 (63)
Liver biopsy, N (%)	70 (51)
Cirrhosis at diagnosis, N (%)	17 (12.5)
Ascites at diagnosis, N (%)	4 (3)
Laboratory values at UDCA initiation, median (IQR)	
ALP (IU/L)	177 (129–252)
ALT (IU/L)	49 (28–63)
AST (IU/L)	42 (28–53)
TB (mg/dL)	0.6 (0.4–0.8)
ALB (mg/dL)	4.3 (4.2–4.5)
PL (×10^9^/L)	261 (211–291)
WB (×10^9^/μL)	6.7 (5.4–7.7)
PT (sec)	11.5 (10.8–12.2)
INR	1 (0.9–1.1)
IgG (mg/dL)	1261 (1015–1484)

Data are expressed as mean and standard deviation or median and interquartile range. ALB, albumin; ALP, alkaline phosphatase; ALT, alanine transaminase; AMA, antimitochondrial antibodies; Anti-gp210, anti-glycoprotein 210; AST, aspartate aminotransferase; BMI, body mass index; dL, deciliter; IgG, immunoglobulin G; INR, international normalized ratio; IQR, interquartile range; IU, International Units; L, liter; μL, microliter; mg, milligram; N, number; PL, platelets; PT, prothrombin time; SD, standard deviations; sec, seconds; TB, total bilirubin; WB, white blood cell count.

**Table 2:** Characteristics of responders and non-responders after 12 months of UDCA treatment (n = 136).

Variables	Responders (N = 110)	Non-Responders (N = 26)	*p* Value
Laboratory values after 12 months of UDCA initiation, median (IQR)			
ALP (IU/L)	130 (99–172)	141 (96–230)	0.001
ALT (IU/L)	16 (25–36)	23 (17–35)	0.004
AST (IU/L)	26 (21–34)	30 (24–44)	0.001
TB (mg/dL)	0.5 (0.3–0.6)	1.2 (1.1–1.3)	<0.001
ALB (mg/dL)	4.3 (4.2–4.5)	4.2 (3.8–4.5)	0.001
PL (×10^9^/L)	255 (213–284)	189.5 (116–261)	0.007
PT (sec)	11.2 (10.9–11.95)	11.7 (11.2–12.5)	0.821
Treatment response, mean (SD)			
Paris criteria, treatment response N (%)	110 (80)	26 (20)	0.014
GLOBE score, treatment response N (%)	111 (82)	25 (18)	0.205
GLOBE score, mean (SD)	−0.82 (1.02)	0.55 (0.95)	<0.001

Data are expressed as mean and standard deviation or median and interquartile range. ALB, albumin; ALP, alkaline phosphatase; ALT, alanine transaminase; AST, aspartate aminotransferase; INR, international normalized ratio; IU, International Units; L, liter; mg, milligram; N, number; PL, platelets; PT, prothrombin time; SD, standard deviations; sec, seconds; TB, total bilirubin; UDCA, ursodeoxycholic acid. UDCA response was defined by the Paris II criteria.

**Table 3:** Changes in UK-PBC score after 12-month UDCA treatment.

UK-PBC Score	Baseline			12 Months after UDCA Treatment		
	Responder	Non-Responder	*p* Value	Responder	Non-Responder	*p* Value
5 y	1.12 (4.22)	3.29 (3.6)	0.000	0.52 (0.43)	2.44 (2.16)	<0.001
10 y	3.17 (8.19)	10.12 (10.03)	0.000	1.7 (1.4)	7.78 (6.5)	<0.001
15 y	5.26 (9.47)	17.27 (15.51)	<0.001	3.13 (2.55)	13.65 (10.8)	<0.001

Data are expressed as mean and standard deviation. UDCA, ursodeoxycholic acid; UK-PBC, United Kingdom–primary biliary cholangitis score; y, years.

**Table 4:** Univariate and multivariable logistic regression analysis for responders to UDCA treatment.

Variable	Univariate			Variable	Multivariate		
	OR	CI 95%	*p* Value		OR	CI 95%	*p* Value
Age	1.02	0.98–1.05	0.305	ALP	1.01	1.00–1.03	0.001
ALP	1.02	1.00–1.05	0.027	AST	1.02	0.99–1.05	0.198
ALT	1.01	0.99–1.02	0.082				
AST	1.02	1.00–1.04	0.006	TB	3.39	0.98–10.3	0.549
TB	9.78	5.82–13.7	<0.001				
ALB	0.14	0.04–0.51	0.002	ALB	2.73	0.50–15.14	0.250
PL	0.99	0.98–0.99	0.005	PL	1.00	0.99–1.01	0.480
GLOBE score	6.64	3.13–14.05	<0.001	GLOBE score	9.30	3.21–24.9	<0.001
UK-PBC score	19.3	5.98–62.5	<0.001	UK-PBC score	32.97	7.44–45.97	<0.001

ALB, albumin; ALP, alkaline phosphatase; ALT, alanine transaminase; AST, aspartate aminotransferase; PL, platelets; TB, total bilirubin; UDCA, ursodeoxycholic acid; UK-PBC, United Kingdom–primary biliary cholangitis score.

## Data Availability

The data can be obtained from the corresponding author upon reasonable request.
